# Obstetrical Nursing for Nurses and Students

**Published:** 1911-01

**Authors:** 


**Obstetrical Nursing for Nurses and Students.** By **Henry Enos Tuley,** A.M., M.D., Professor of Obstetrics, Medical Department University of Louisville. With seventy-three illustrations. Second edition. John P. Morton & Company, Publishers, Louisville, Ky. 1910. (Cloth, $1.50.)

The material for this manual has been carefully selected and conveniently arranged. A perusal of its contents will be of great value to any nurse or medical student, and a careful reading of the work will do the general practitioner of medicine no harm. In a necessarily brief, but in a very clear manner, the various details needed in cases of labor are considered and suggestions made as how to improvise what is not at hand but required in the sick-room. It is not too much to expect that one who nurses should know, or become familiar with the contents of this book. This edition is practically new as the original plates of the first were destroyed. J.A.R.

